# Transcriptomic analyses reveal regulatory plasticity and metabolic reprogramming underlying genotype-specific microspore embryogenesis in wheat

**DOI:** 10.1007/s00299-026-03731-x

**Published:** 2026-02-04

**Authors:** Hai Ying Yuan, Yunfei Jiang, Palak Kathiria, Venkatesh Bollina, Yifang Tan, Jean L. Enns, Alison M. R. Ferrie, Sateesh Kagale

**Affiliations:** 1 Aquatic and Crop Resource Development Research Centre, National Research Council Canada, Saskatoon, SK S7N 0W9 Canada; 2 Department of Plant, Food, and Environmental Sciences, Faculty of Agriculture, Dalhousie University, Truro, NS B2N 5E3 Canada; 3 INARI, Cambridge, MA 02139 USA; 4 Nuseed, Sacramento, CA 95605 USA

**Keywords:** Wheat, Microspore embryogenesis, RNA seq, Transcription factor, Machine learning, Homoeolog expression

## Abstract

**Key message:**

Embryogenic efficiency in wheat microspores is driven by epigenetic regulation, homoeolog expression bias, and genotype-specific genomic variation, with coordinated remodeling of metabolic pathways and cell-wall dynamics establishing a favourable cellular environment.

**Abstract:**

Microspore embryogenesis is a process in which immature male gametophytes are induced to form embryo-like structures that can regenerate into doubled haploid (DH) plants following chromosome doubling. By producing complete homozygosity in a single generation, DH technology accelerates cultivar development and is particularly valuable for breeding resilient crops. However, bread wheat remains largely recalcitrant to microspore embryogenesis, with strong genotype dependence limiting its broad application in breeding programs. Here, we performed a comparative transcriptomic time-course in two spring wheat cultivars with contrasting embryogenic responses: Nanda (highly responsive) and Sadash (recalcitrant). Dynamic gene expression reprogramming was observed during embryogenesis, with Nanda exhibiting enrichment of biological processes associated with epigenetic regulation, including nucleosome assembly, chromatin remodeling, and chromatin organization. In addition, genes related to stress perception, hormonal signaling, cytoskeletal and cell wall dynamics, and metabolic pathways showed coordinated expression changes, collectively characterizing the transcriptional landscape associated with successful microspore embryogenesis. Differentially expressed gene (DEG) hotspots identified structural variation underlying the divergent responses between genotypes. Machine learning highlighted potential biomarkers, notably a histone deacetylase gene TRAESCS1D02G454400 located within a DEG-enriched region. Subgenome-specific analysis revealed pronounced suppression of B-subgenome homoeologs in Sadash, 65% of which overlapped with DEGs from the genotype comparison. These findings highlight the role of epigenetic regulation, homoeolog expression bias, and genotype-specific genomic variation in determining embryogenic efficiency. Importantly, these conclusions are based on transcriptomic associations and require functional validation, while providing candidate molecular targets and biomarkers to overcome recalcitrance and enhance the utility of microspore embryogenesis in wheat DH breeding.

**Supplementary Information:**

The online version contains supplementary material available at 10.1007/s00299-026-03731-x.

## Introduction

Microspore embryogenesis, or androgenesis, is a developmental reprogramming process in which immature male plant gametophytes (micropores, the immature form of pollen grains) are diverted from their normal pollen development pathway and induced to form embryo-like structures (ELSs). These structures can subsequently be regenerated into doubled haploid (DH) plants through whole genome reduplication (Seifert et al. 2016; Żur et al. 2019; Gajecka et al. 2021). The ability to produce completely homozygous lines in a single generation makes androgenesis a powerful tool in plant breeding, reducing the time and cost required for cultivar development (Ferrie et al. 1995). Androgenesis can be divided into three phases: Phase I, the attainment of embryogenic competence through stress-mediated cell redifferentiation, which is the prerequisite for embryoid development; Phase II, the development of multicellular structures (MCS, or proembryos) confined within the exine wall; and Phase III, pre-embryos being released from the exine wall and reaching the mature embryo stage (Zheng 2003; Maraschin et al. 2005; Testillano 2019).

Androgenesis usually requires a stress pre-treatment (such as cold, heat, carbohydrate, or nitrogen starvation), which blocks gametophytic development by arresting the expression of gamete-specific genes and induces embryogenic development by cell reprogramming (Harada et al. 1986; Zheng 2003; Shariatpanahi et al. 2006). Subsequent success of embryogenesis depends on a favourable in vitro culture environment, with medium components including antioxidants, antibiotics, arabinogalactan proteins, and epigenetic modulators shown to improve androgenetic efficiency in wheat and related species (Wang et al. 2019; Hale et al. 2022).

Bread wheat is one of the most important cereal crops worldwide. However, most wheat cultivars are recalcitrant to androgenesis (Seifert et al. 2016). Current limitations include low efficiency of haploid embryo induction, poor regeneration rates, and frequent production of albino plants (Soriano et al. 2013). These bottlenecks restrict the utility of microspore culture for large-scale DH production in wheat breeding programs. The process and effectiveness of androgenesis are shaped by a complex interplay of genetic, epigenetic, and environmental factors, involving transcriptional reprogramming, developmental plasticity, and subgenome-specific regulation in polyploid wheat. However, the molecular basis of these processes, and particularly the reasons why some genotypes respond efficiently while others remain recalcitrant, remains poorly understood.

Gene expression and transcriptomic studies in barley (*Hordeum vulgare*), canola (*Brassica napus*) and sweet pepper (*Capsicum annuum*), and somatic embryogenesis (SE) in maize (*Zea mays*) have provided valuable insights into the dynamics of gene expression during embryogenesis (Malik et al. 2007; Salvo et al. 2014; Seifert et al. 2016; Bélanger et al. 2018; Cheng et al. 2020). In wheat, RNA-seq analysis has revealed transcriptional shifts in a responsive winter cultivar (“Svilena”), identifying stage-specific changes during microspore embryogenesis (Seifert et al. 2016). Yet, no study has compared spring wheat genotypes with contrasting embryogenic responses, nor investigated subgenome-specific contributions and genomic regions underlying efficiency differences.

Here, we conducted a comprehensive transcriptomic time-course of microspore embryogenesis in two spring wheat genotypes with contrasting androgenetic responses: Nanda, a highly responsive cultivar, and Sadash, a relatively recalcitrant genotype (Sadasivaiah et al. 2000, 2011). We profiled gene expression at three key stages, including uninucleate microspores (Day 0), proembryos (Day 7), and embryo-like structures (Day 21), and integrated differential expression, functional enrichment, subgenome-specific analyses, and machine learning approaches. Our study (i) uncovers causative biological processes underlying embryogenic induction and progression, (ii) identifies molecular networks and candidate regulators distinguishing high- and low-responsive genotypes, (iii) reveals homoeolog expression dynamics and DEG-enriched genomic regions associated with embryogenesis, and (iv) highlights potential biomarker genes for improving androgenesis efficiency. These findings highlight transcriptional patterns associated with embryogenic success in wheat and may inform future strategies to address recalcitrance in DH breeding pipelines.

## Materials and methods

### Plant material and sample collection

Two spring wheat genotypes with contrasting androgenesis responses were evaluated—Nanda (Sadasivaiah et al. 2000), which is highly embryogenic, and Sadash (Sadasivaiah et al. 2011), which shows limited responsiveness. Seeds were sown in 8-inch pots filled with Sunshine LA4 (Sungro Horticulture, AB, Canada) with 4.5 g slow-release Nutricote^®^ fertilizer, 14-13-13. Plants were thinned to three plants per pot after seedling emergence. They were grown under the conditions of 18/6 h photoperiod, 20/18 °C day/night temperatures, and 350–420 μmol/m^2^/s light intensity in a growth chamber (PGW40, Conviron, Winnipeg, MB, Canada) at the Aquatic and Crop Resource Development Research Centre (ACRD), National Research Council Canada (NRC) in Saskatoon. Spikes were harvested when microspores reached the mid- to late-uninucleate stage (staged using acetocarmine staining), and microspores were extracted after spikes were exposed to three weeks of 4 °C cold pre-treatment. Procedures of microscope isolation and culture, including compositions of culture media, were previously described (Wang et al. 2019), and the maltose gradient centrifugation used in the step improved the uniformity of microspores. Samples were collected from three main stages of androgenesis—microspores at Day 0, proembryos at Day 7, and ELSs at Day 21, for both cultivars.

### RNA extraction and sequencing

The RNAqueous™-Micro Total RNA Isolation Kit (Thermo Fisher Scientific, Waltham, MA, USA) was used to extract total RNA from pooled microspores. The RNA yield and purity were assessed using Nanodrop 1100 (Thermo Fisher Scientific, Waltham, MA, USA), and the RNA quality /integrity was evaluated using an Agilent 2100 Bioanalyzer (Agilent Technologies Inc., Santa Clara, CA, USA). 18 RNA-seq libraries [(two cultivars × three developmental stages) × three biological replicates] were prepared using the standard Illumina TruSeq RNA library kits following the manufacturer’s instructions (Illumina, San Diego, CA, USA). Paired-end sequencing (2 × 150 bp) was performed on an Illumina HiSeq2500 platform at ACRD NRC in Saskatoon, Canada. Sequencing adapters, low-quality reads and reads shorter than 75 bp were trimmed using Trimmomatic (v0.39) with default settings (Bolger et al. 2014). Filtered reads for each sample were aligned to the wheat reference genome (IWGSC v1.1) (International Wheat Genome Sequencing Consortium et al. 2018) using STAR (v2.7.5a) (Dobin et al. 2013). Relative gene abundance was estimated using the IWGSC v1.1 annotation and the RSEM (v1.3.3) algorithm (Li and Dewey 2011). We excluded low-confidence (LC) gene models of IWGSC v1.1 from the downstream analyses to eliminate potential confounding effects from pseudogenes and low-quality gene models.

### Transcriptomic analysis

The iDEP workflow (version 2.01) was used for the transcriptomic gene expression analysis (Ge et al. 2018). RNA-seq profiling from three time points/ developmental stages (Day 0, Day 7 and Day 21), with three biological replicates per time point across both genotypes, resulted in 18 samples. Raw read counts were normalized using the CPM (counts per million) function in edgeR (version 4.0) (Robinson et al. 2010; Chen et al. 2016). Genes with a CPM value of ≥ 1 in at least one of the 18 samples were considered expressed and retained for downstream analysis. 62,692 genes, out of 107,891 genes from the IWGSC v1.1 annotation, passed the filter and were retained for downstream analysis. Normalized gene read counts were log-transformed for principal component analysis (PCA). PCA was performed using the prcomp function in R (R Core Team 2022).

Differentially expressed genes (DEGs) were analyzed for developmental transitions (Day 0 to Day 7 and Day 7 to Day 21 in both genotypes), and genotype comparison at the same stages using DeSeq2 (Love et al. 2014) within the iDEP workflow. Genes with the adjusted *p* value less than 0.01 and |log_2_FC| ≥ 1 were considered differentially expressed. The contrasting groups included (1) Nanda_Day7 vs Nanda_Day0 (ND7_D0), (2) Nanda_Day21 vs Nanda_Day7 (ND21_D7), (3) Sadash_Day7 vs Sadash_Day0 (SD7_D0), (4) Sadash_Day21 vs Sadash_Day7 (SD21_D7), (5) Nanda_Day0 vs Sadash_Day0 (NS_D0), (6) Nanda_Day7 vs Sadash_Day7 (NS_D7), (7) Nanda_Day21 vs Sadash_Day21 (NS_D21). The comparisons (1)-(4) reflected the developmental switch (effect of timing) in Nanda (1–2) and Sadash (3–4); while the comparisons (5–7) reflected the genotype effect at the same developmental stages. Mercator pipeline 4, version 7.0 (Schwacke et al. 2019; Bolger et al. 2021) was used to annotate DEGs from the wheat reference genome (IWGSC v1.1) using the longest peptide sequences.

### Homoeolog expression analysis

To understand the dynamics of homoeolog expression during microspore embryogenesis and the effect on the efficiency of the process, we used the gene triad list that had a 1:1:1 correspondence across the three homoeologous subgenomes of wheat (Sun et al. 2023). There are 55,221 genes from the list, with 18,407 from each of the A, B and D subgenomes. 62,692 expressed genes from the transcriptomic analysis were used in the homoeolog expression analysis. A triad was considered expressed if the sum of the expression from three homoeologs across A, B and D subgenomes was larger or equal to 5 TPM (≥ 5 TPM) in any of the six developmental stages between Nanda and Sadash. We then standardize the relative expression of each homoeolog across the triad using the method of Ramírez-González et al. (2018). Briefly, the relative expression of A subgenome homoeolog was derived using the TPM value of A subgenome homoeolog divided by the sum of TPM values from A, B and D subgenome homoeologs. Using the same approach, we calculated the normalized relative expression of each homoeolog from each subgenome.

Seven categories of expression patterns used in Ramírez-González et al. (2018) were adopted here. Triad expression pattern was assigned: A dominant if the relative expression of A subgenome homoeolog > 60 and the relative expression of B and D subgenome homoeologs ≤ 20; B dominant and D dominant were assigned using a similar approach. A suppressed if the relative expression of A subgenome homoeolog < 20 and the relative expression of B and D subgenome homoeologs < 80; the same for B suppressed and D suppressed. The rest of the triads not belonging to these six expression patterns were referred to as balanced. R package ggtern and ggplot2 were used to visualize homoeolog expression patterns across development stages and between genotypes (Wickham 2016; Hamilton and Ferry 2018).

### Gene ontology (GO) enrichment analysis

First, to explore functional mechanisms underlying successful developmental transitions during androgenesis, Gene Ontology:Biological Process (GO:BP) enrichment analysis was conducted on upregulated genes during developmental transitions in Nanda (ND7_D0 and ND21_D7) using g:Profiler (version e111_eg58_p18_f463989d) (Kolberg et al. 2023). Similarly, downregulated genes were also used for GO enrichment analysis using g:Profiler. Annotated genes from the wheat reference genome (IWGSC v1.1) were used as the background for the analysis. g:SCS algorithm was used for computing multiple testing corrections of p-values with a significance threshold of 0.05. GO:BP terms with minimum size of 10 and maximum size of 2000 were used for the enrichment analysis. Enrichment results of both upregulated and downregulated genes from the same contrasting group were combined and then used as network construction and visualization inputs in Cytoscape (version 3.10.1) (Shannon et al. 2003). gProfiler enrichment cutoff of 0.01 was used as the node cutoff. The similarity at 0.375 with a combined constant at 0.5 was used as the edge cutoff for the enrichment map construction in Cytoscape.

Second, to underpin molecular mechanisms contributing to Nanda's higher androgenesis efficiency compared to Sadash, upregulated and downregulated genes in Nanda compared to Sadash at the same developmental stages (NS_D0, NS_D7, and NS_D21) were used for GO:BP enrichment analysis using g:Profiler with the same settings as shown in the previous section. Cytoscape was further used to construct and visualize the enrichment networks with gProfiler enrichment cutoff of 0.05 used as the node cutoff and a similarity of 0.375 with a combined constant of 0.5 used as the edge cutoff.

### Transcription factors in microspore embryogenesis

To understand the trans-regulatory landscapes in the developmental transition of microspore embryogenesis and the differences in these regulatory elements contributing to Nanda’s high embryogenic efficiency compared to Sadash, Mercator pipeline 4, version 7.0 (Schwacke et al. 2019; Bolger et al. 2021) was used to annotate TFs from the wheat reference genome (IWGSC v1.1) using the longest peptide sequences. Mercator-identified TFs were then used to examine their presence in the respective DEGs during developmental transitions in Nanda (ND7_D0 and ND21_D7) and genotype comparisons (NS_D0, NS_D7, and NS_D21).

### Expression over-representation analysis of genomic regions

PREDA package (version 1.42.0) was used to scan the genome for genomic regions with the overrepresentation of differentially expressed genes (Ferrari et al. 2011; Ferrari 2022). DEGs in Nanda compared to Sadash at the same developmental stages (NS_D0, NS_D7, and NS_D21) were used for the analysis, and all annotated genes from the wheat reference genome (IWGSC v1.1) (International Wheat Genome Sequencing Consortium et al. 2018) were used as the background. PREDA analysis was conducted using Log2 fold changes of genome-wide gene expression for each contrast group. Local kernel regression smoothing was applied as the smoothing method. Genomic regions with the overrepresentation for each comparison were derived from a Student’s *t*-test, and an FDR-corrected *p*-value (< 1e−05) was used to define the significantly enriched genomic regions.

### Machine learning classification of RNA-seq data and feature selection

R package MLseq (Goksuluk et al. 2019) was used to classify RNA-seq data using raw read counts from 62,692 expressed genes identified in the earlier transcriptomic analysis step. The data used in MLseq was (raw read count + 1) to prevent zero division problems during the model training process and was split into two parts: 60% as the training sets, while the rest 40% as the test sets. Data was normalized using the DeSeq median ratio normalization provided in the package, and Voom-based Nearest Shrunken Centroids (VoomNSC) was used as the classifier to train the model. The model was trained using two sets of parameters: fivefold cross-validation repeated 2 times, and fivefold cross-validation repeated 10 times. Model performance was evaluated using metrics, such as accuracy rate, sensitivity and specificity, included in the MLseq package. Then, the model was used to select significant features/genes as possible biomarkers.

## Results

### High androgenetic efficiency in Nanda

**Fig. 1:**
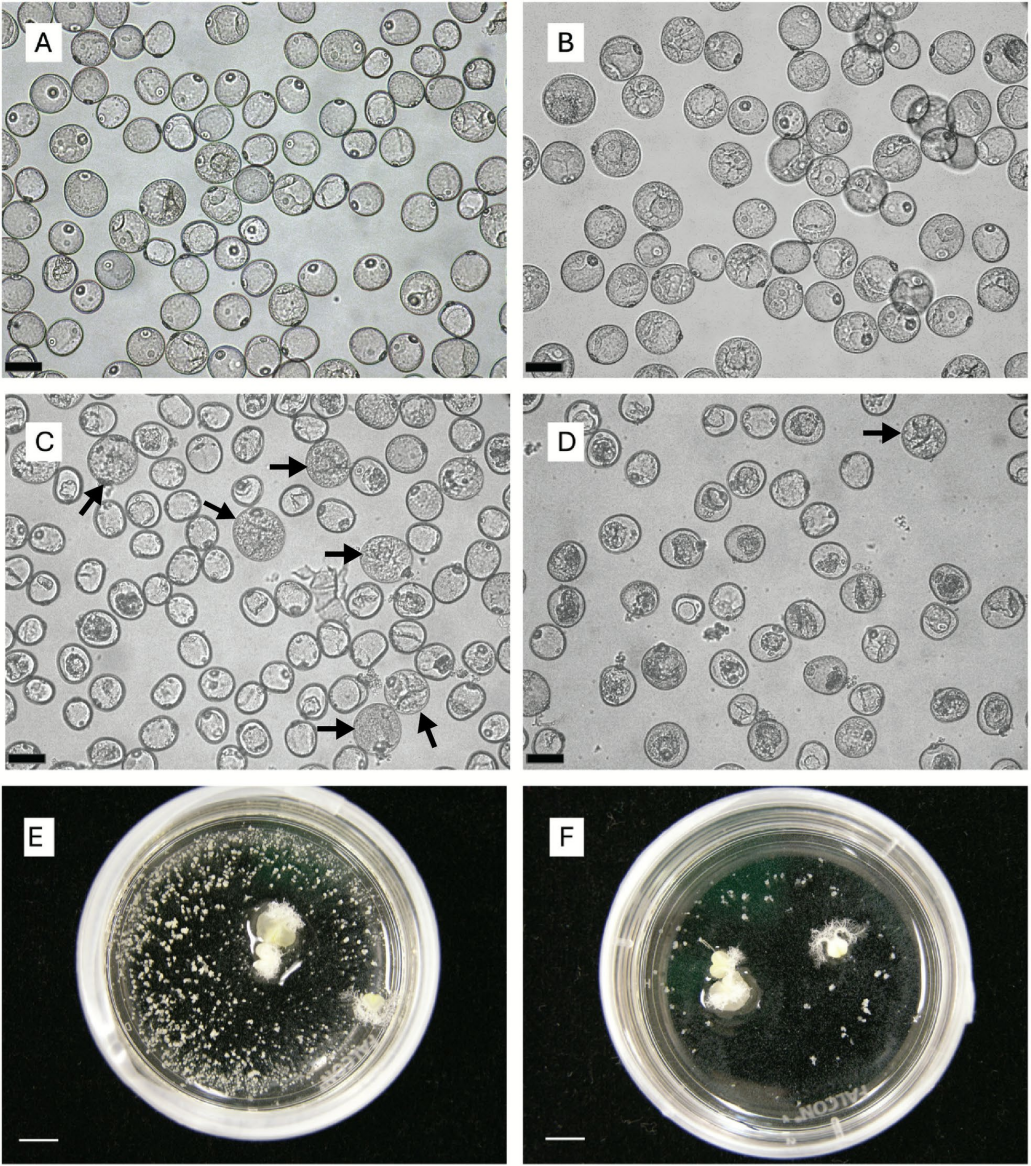
Difference in microspore embryogenesis in two spring wheat genotypes, Nanda (**A**, **C** and **E**) and Sadash (**B**, **D** and **F**). Images show three main stages of microspore embryogenesis—microspores at Day 0 (**A** and **B**), proembryos at Day 7 (**C** and **D**), and embryo-like structures (ELSs) at Day 21 (**E** and **F**). Proembryos at Day 7 were marked with arrows (**C** and **D**), while ELSs contain both large and smaller, less well-developed structures (**E** and **F**). Scales for **A**–**D** 40 μm and scales for **E**, **F** 5 mm

The efficiency of androgenesis is a critical determinant of successful DH production in cereal crops, as it is directly related to the frequency of embryo formation and subsequent plant regeneration. In the present study, comparative analysis of two wheat genotypes, Nanda and Sadash, revealed striking differences in their androgenetic response under microspore culture conditions. In both genotypes, the development of multicellular structures, known as pro-embryos, occurs approximately seven days after the initiation of microspore culture (Fig. 1). These early-stage structures remained enclosed within the exine and continued to develop over time. By the second week, distinct microspore-derived ELS had begun to form, which further expanded in size by day 21 (Fig. 1). Nanda produced a significantly higher number of embryos per spike than Sadash. On average, 1002 ELS per spike were produced in Nanda, whereas Sadash produced only 25 ELS. The efficiency of green plant regeneration also varied greatly between the two genotypes. In Nanda, within the regenerated plants, 51% were green plants, while the remaining 49% albino plants. In contrast, only 2% of regenerants in Sadash were green plants, and the remaining 98% were albino plants. These observations establish Nanda as a highly responsive genotype, with markedly greater ELS formation and green plant regeneration than Sadash.

### Transcriptomic dynamics during androgenesis and genotypic variation

**Fig. 2:**
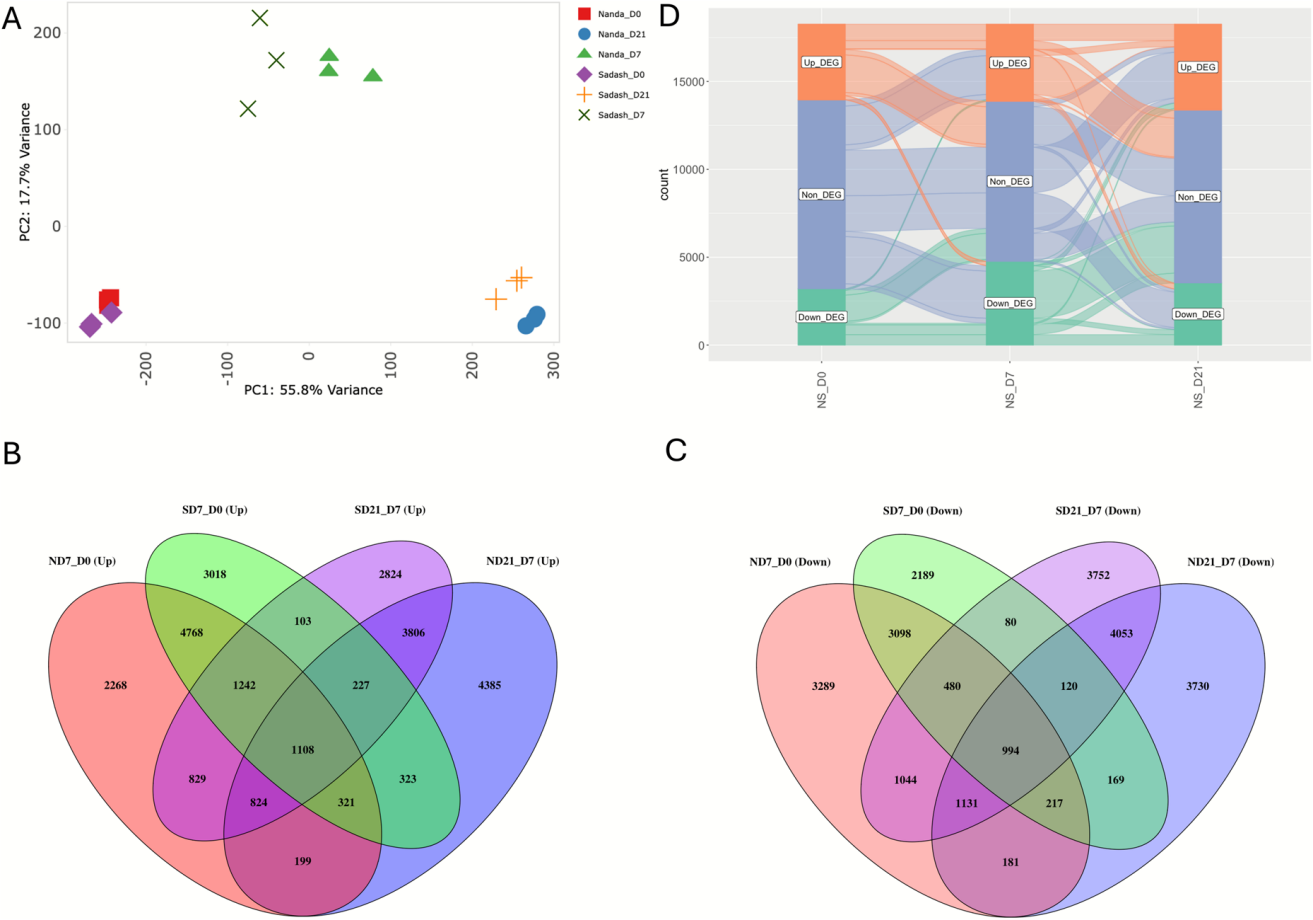
Global transcriptomic relationships among stages of microspore embryogenesis in two spring wheat genotypes, Nanda and Sadash. **A** Principle component (PCA) plot of RNA-seq data illustrating sample-to-sample distances (within and between genotypes across three developmental stages) on the first two principal components. **B** Venn diagram illustrating distinct and overlapping up-regulated genes between stages of microspore embryogenesis within Nanda and Sadash. **C** Venn diagram illustrating distinct and overlapping down-regulated genes between stages of microspore embryogenesis within Nanda and Sadash. **D** Alluvial plot illustrating up-regulated genes (Up_DEG), non-differentially expressed genes (Non_DEG) and down-regulated genes (Down_DEG) from the comparison between Nanda and Sadash across three developmental stages. DEGs are defined as |log_2_FC| ≥ 1 and FDR < 0.01

To investigate transcriptional changes associated with developmental transitions during androgenesis and the distinct embryogenic capacities of Nanda and Sadash, we analyzed differentially expressed genes (DEGs; |log_2_FC| ≥ 1 and adjusted *p* < 0.01) across developmental stages and genotypes. Before the DEG analysis, hierarchical clustering analysis was performed to visualize expression patterns among all 18 samples from both Nanda and Sadash (Additional file 1: Fig. S1). High transcriptome data quality was confirmed through strong correlation among biological replicates (Additional file 1: Fig. S2A). Principal Component Analysis (PCA) of log-transformed normalized gene read counts demonstrated tight clustering of replicates by both development stage and genotype, indicating consistent transcriptomic responses during androgenesis (Fig. 2A). PCA further revealed a clear separation between the development stages and the two genotypes, with the first two components (PC1 and PC2) explaining 73.5% of total variance. Collectively, the first five principal components accounted for over 90% of the observed variation (Additional file 1: Fig. S2B).

Differential expression analyses were performed to assess transcriptional dynamics during two key developmental transitions in Nanda (N) and Sadash (S): Day 7 vs. Day 0 (ND7_D0 and SD7_D0) and Day 21 vs. Day 7 (ND21_D7 and SD21_D7) (Fig. 2B and C, Additional file S1). Nanda showed a robust transcriptional response. With 21,993 DEGs identified in ND7_D0, of which 53% were upregulated. In contrast, Sadash exhibited a reduced response, with 18,457 DEGs in SD7_D0, and a slightly higher proportion of upregulated genes (60%). Furthermore, 51% of 21,788 DEGs from the ND21_D7 group were upregulated as compared to 48% of 22,617 DEGs from the SD21_D7 group.

To explore genotype-specific gene expression differences, pairwise comparisons were made between Nanda and Sadash at each stage using low embryogenic Sadash as a reference (NS_D0, NS_D7, NS_D21; Fig. 2D; Additional file 1: Fig. S2C and S2D). The number of DEGs identified was notably lower in these comparisons, with 7517, 9,156, and 8,426 DEGs on Day 0 (NS_D0), Day 7 (NS_D7), and Day 21 (NS_D21), respectively. Upregulation rates remained relatively consistent, with 57- 58% of DEGs upregulated in NS_D0 and NS_D21, and a slightly lower rate (48%) in NS_D7. These results highlight distinct transcriptomic programs governing microspore embryogenesis, with Nanda exhibiting a more dynamic and extensive transcriptional response than Sadash, consistent with its higher androgenetic efficiency.

### Homoeolog expression dynamics during microspore embryogenesis

Given the hexaploid nature of wheat, with A, B, and D subgenomes, we investigated how homoeolog-specific expression patterns contribute to differences in embryogenic potential between Nanda and Sadash. A triad was considered expressed if the sum of the expression from three homoeologs across A, B and D subgenomes was ≥ 5 TPM in any of the six developmental stages between Nanda and Sadash (Nanda: Day 0, 7 and 21; Sadash: Day 0, 7 and 21). This threshold resulted in 12,813 expressed triads during microspore embryogenesis (Additional file S2), corresponding to 61.3% of expressed genes and 69.6% of triads from the reference list. This threshold also captured both stage-specific triads and those with consistent expression across stages.

**Fig. 3:**
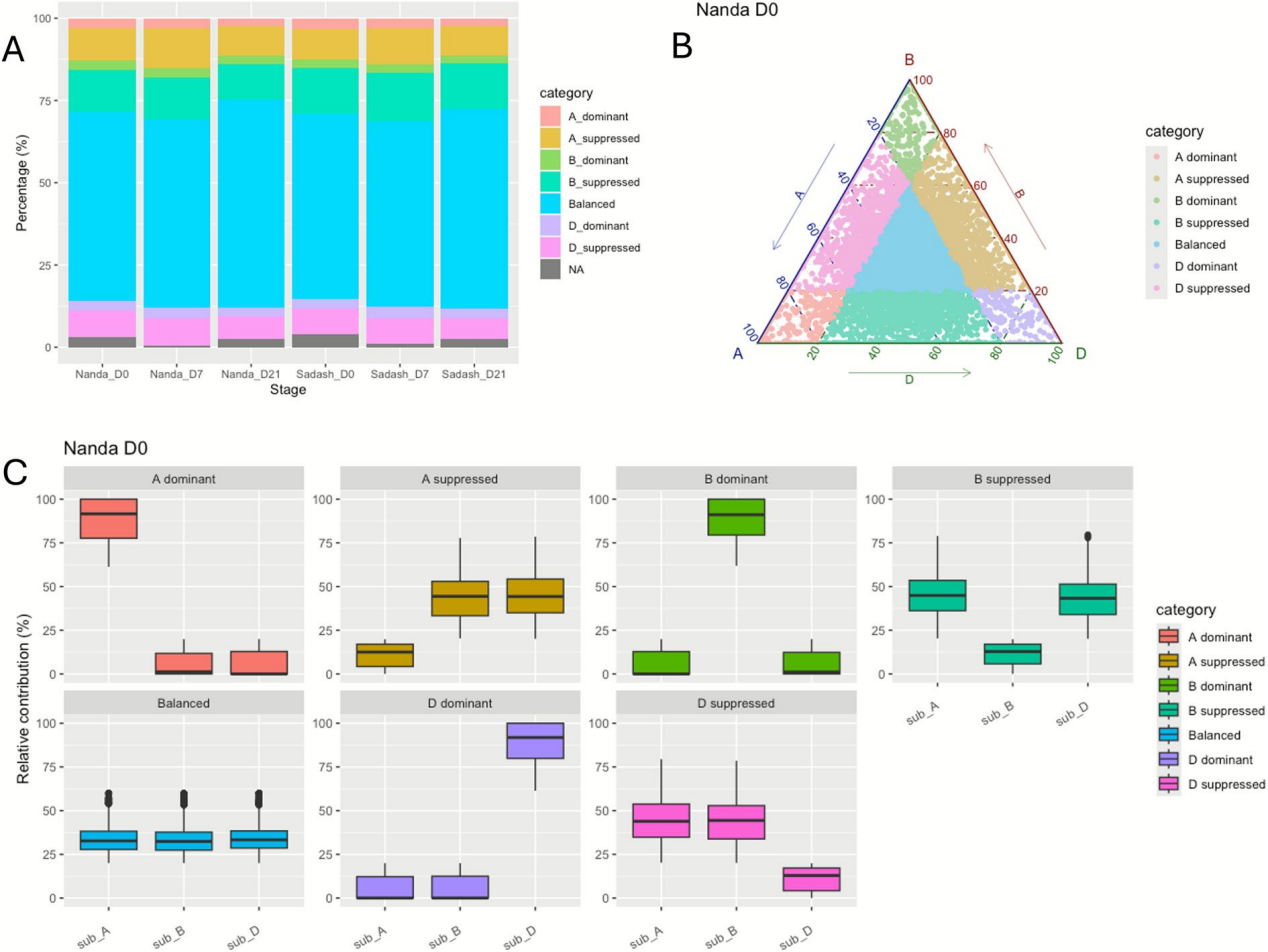
Homoeolog expression dynamics during microspore embryogenesis. **A** Proportion of triads in each category of homoeolog expression bias across different stages in Nanda and Sadash. **B** Ternary plot illustrating the relative expression abundance of 12,813 syntenic triads on Day 0 of Nanda microspore embryogenesis. Each dot represents a triad with **A**, **B**, and **D** coordinates, indicating the relative contribution of each homoeolog to the overall expression of the triad. **C** Box plots illustrating the relative contribution of each subgenome based on triad assignment to the seven categories on Day 0 of Nanda microspore embryogenesis

In general, most triads belonged to the balanced category across developmental stages in both Nanda and Sadash, with similar percentages on Day 0 and Day 7 and a higher percentage on Day 21 (Fig. 3A). Among biased categories, single-homoeolog-suppressed triads were more common than single-homoeolog-dominant triads in both genotypes, with the former ranging from 6.5 to 14.7% and the latter from 2.3 to 3.3% (Fig. 3A). Percentages of single-homoeolog-dominant triads remained similar across subgenomes, whereas B-suppressed triads were consistently most abundant and D-suppressed triads least abundant. Relative expression abundance of the 12,813 expressed triads and the relative contribution of each subgenome to seven triad expression categories in Nanda at Day 0 are shown in Fig. 3B and C, with additional stages shown in Additional file 1: Fig. S3.

Homoeolog expression patterns remained unchanged in 52.4% of expressed triads across developmental stages in Nanda and 53.7% in Sadash. Among these, the majority of balanced triads stayed balanced, accounting for 71.7%, 72.2%, and 65.3% for Days 0, 7, and 21 in Nanda, and 73.0%, 72.8%, and 67.6% in Sadash. By contrast, dominant and suppressed triads were less stable: only 18.9–27.4% of dominant triads and 26.3–40.4% of suppressed triads remained unchanged in Nanda. Sadash showed a similar percentage of stable dominant triads but a higher proportion of stable suppressed triads, particularly B-suppressed triads. Across three stages, an average of 45.1% of B-suppressed triads remained unchanged in Sadash, compared to only 36.2% in Nanda. Comparing with stable B-suppressed triads from Nanda, we identified 480 unique stable B-suppressed triads in Sadash, with more than 300 of their B-subgenome genes appearing in DEG lists across NS_D0, NS_D7, and NS_D21, where they were upregulated (Additional file S2). These findings highlight homoeolog expression bias, particularly B-subgenome suppression, as an important factor underlying differences in embryogenic potential between Nanda and Sadash, providing a subgenomic dimension to transcriptomic regulation of androgenesis.

### Mechanisms underlying androgenetic progression

To explore the molecular basis of successful developmental transitions during androgenesis, we performed functional Gene Ontology: Biological Process (GO:BP) enrichment analysis (FDR < 0.05) on DEGs identified across key developmental transitions in Nanda. During the early transition from Day 0 to Day 7 (ND7_D0), up-regulated GO:BP categories outnumbered down-regulated ones, highlighting extensive transcriptional reprogramming during the switch from microspore to embryogenic development (Additional file 1: Fig. S4A; Additional file S3). The significantly up-regulated GO: BP terms included those related to chromatin and cytoskeletal organization, such as nucleosome assembly and organization, chromosome remodelling, and microtubule-based processes, including assembly, organization, and movement. Metabolic processes, particularly those involving polysaccharide metabolism, glutathione metabolism, and amino acid pathways like glutamine metabolism, were also significantly enriched. Notably, several signalling pathways were also activated, including cellular response to various stimuli (chemical stimulus, endogenous stimulus, abiotic stimulus, and organic/inorganic substance) and hormone-mediated signalling (e.g., auxin-activated pathways). In contrast, downregulated GO terms in ND7_D0 were associated with RNA modification, ncRNA and tRNA metabolism, phospholipid metabolism, and transmembrane transport including metal ion, inorganic ion, and monoatomic ion transport. Downregulation of protein ubiquitination and starch biosynthesis, a hallmark of the developmental shift from gametophytic to embryogenic fate, was also observed.

**Fig. 4:**
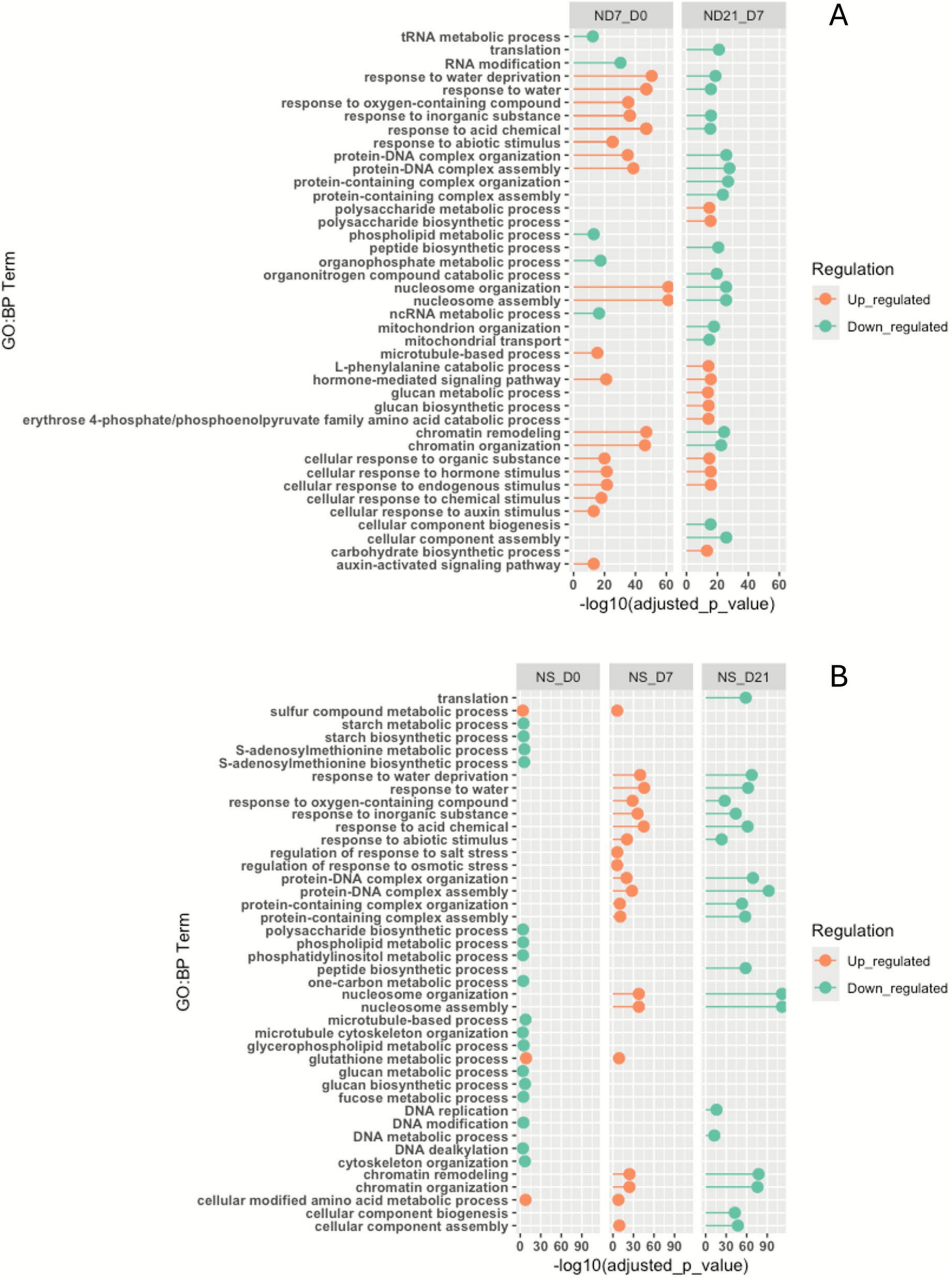
Gene ontology enrichment analysis of DEGs. (A) Lollipop plot illustrating the top 30 enriched GO:BP terms between stages of Nanda microspore embryogenesis. (B) Lollipop plot illustrating the top 20 enriched GO:BP terms from the Nanda and Sadash comparisons across three developmental stages. Orange shows terms enriched from up-regulated genes, while green shows terms enriched from down-regulated genes

During the second transition from Day 7 to Day 21 (ND21_D7), many regulator patterns persisted, consistent with continued embryogenic progression (Additional file 1: Fig. S4B). Key upregulated GO:BP categories included polysaccharide metabolism, hormone-mediated signalling, and cellular responses to internal and external stimuli. Unique to this stage were enriched terms such as cinnamic acid and L-phenylalanine metabolic processes, and intracellular signal transduction. Conversely, uniquely downregulated terms included catabolic pathways for organonitrogen compounds, sugars like galactose and trehalose, and zygotic developmental processes such as double fertilization and endosperm formation, further supporting the switch to microspore-derived embryogenesis. Interestingly, starch biosynthesis, previously downregulated in ND7_D0, showed a reversal in trend. An overview of the top 30 enriched GO:BP terms across both transitions is presented in Fig. 4A. Notably, chromatin-related processes such as nucleosome assembly and chromosome remodelling flipped from strong upregulation during ND7_D0 to downregulation during ND21_D7, reflecting a dynamic reorganization of cellular machinery during androgenetic progression.

### Mechanisms underlying higher embryogenic efficiency in Nanda

To elucidate the molecular mechanisms contributing to higher embryogenic efficiency in Nanda compared to Sadash, GO:BP enrichment analysis was performed on DEGs from genotype comparisons across three developmental stages (NS_D0, NS_D7, and NS_D21). The analysis revealed distinct stage-specific patterns of enriched biological processes differentiating the two genotypes. At the initial stage (Day 0, NS_D0), only a few GO:BP terms were upregulated in Nanda, including glutathione metabolic process and negative regulation of protein phosphorylation. The majority of enriched GO terms were down-regulated, involving key developmental and metabolic pathways such as starch biosynthesis, glucan metabolism, monosaccharide metabolism and microtubule cytoskeleton organization (Additional file 1: Fig. S5A). Processes related to chromatin organization were also notably down-regulated, suggesting delayed transcriptional activation prior to androgenesis induction.

A pronounced shift was observed by Day 7 (NS_D7), with enriched GO terms upregulated in Nanda (Additional file 1: Fig. S5B). Key regulatory processes, including nucleosome assembly, chromosome remodelling, and chromatin organization were among the top upregulated terms. Signalling pathways such as responses to abiotic stimuli, inorganic substance, oxygen-containing compounds, and hormone-mediated pathways were also enriched. Several metabolic processes, including carboxylic acid and organic acid metabolism showed increased activity. Notably, the glutathione metabolic process remained consistently upregulated across both early stages (NS_D0 and NS_D7). Only a limited number of processes, including DNA-templated transcription initiation and ion transmembrane transport, were down-regulated at this stage.

By Day 21 (NS_D21), when embryogenic divergence between the two genotypes was most pronounced, a broader range of GO terms was enriched in Nanda (Additional file 1: Fig. S5C). Upregulated processes included aromatic amino acid, cellulose and glucan metabolism, aligning with the increased number of microspore-derived embryos in Nanda. In contrast, signalling pathways such as the response to abiotic stimuli, inorganic substances, and oxygen-containing compounds were downregulated. Key developmental regulators, including chromatin remodelling, nucleosome assembly, and protein-DNA complex organization, also showed strong downregulation, suggesting a shift from transcriptional regulation to metabolic execution during late embryogenesis. Additionally, RNA processing and metabolism of pyruvate and L-proline were among the down-regulated GO terms. A summary of the top 20 enriched GO:BP terms across the three stages is shown in Fig. 4B. NS_D7 and NS_D21 shared more enriched GO categories than NS_D0, though many were oppositely regulated. Unsurprisingly, several enriched GO terms associated with Nanda's developmental transitions also appeared among the top enriched terms distinguishing it from Sadash, reinforcing their likely role in successful androgenesis. The stage-specific activation of chromatin remodeling, metabolic, and hormone-mediated processes in Nanda correlates with its enhanced ELS formation, providing mechanistic insights into genotype-dependent androgenetic responses.

### Transcription factors associated with microspore embryogenesis

**Fig. 5:**
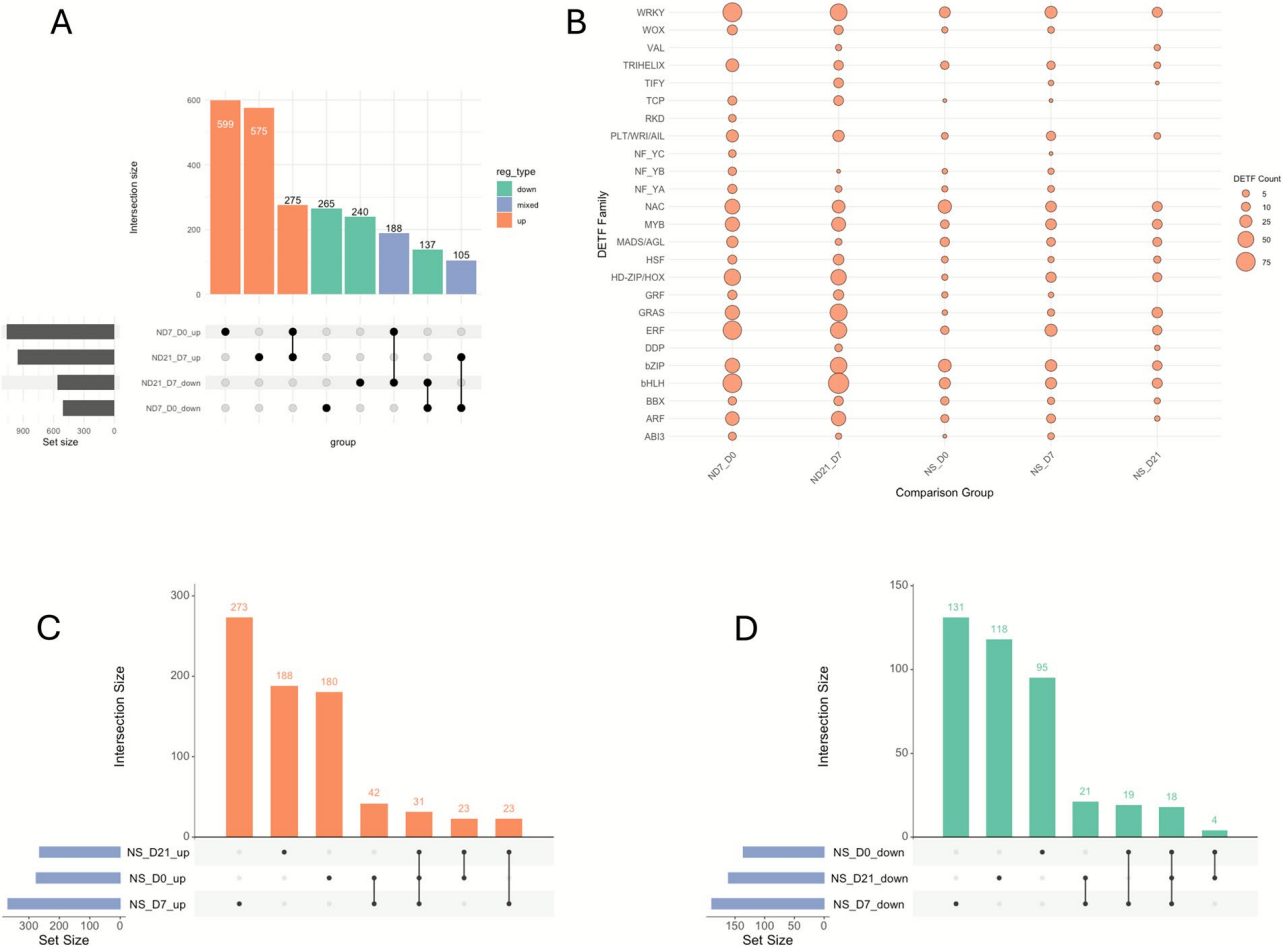
Analysis of differentially expressed transcription factors (DETFs). **A** Upset plot illustrating distinct and overlapping DETFs between stages of Nanda microspore embryogenesis. **B** Dot plot illustrating TF families having the most upregulated TFs between stages of Nanda microspore embryogenesis and across three developmental stages based on the comparison between Nanda and Sadash. **C** UpSet plot illustrating distinct and overlapping upregulated TFs from the comparison between Nanda and Sadash across three developmental stages. **D** UpSet plot illustrating distinct and overlapping downregulated TFs from the comparison between Nanda and Sadash across three developmental stages

Transcription factors (TFs) play key roles in regulating gene expression during developmental processes, including microspore embryogenesis (Méndez-Hernández et al. 2019; Tian et al. 2020; Verma et al. 2022; Yuan et al. 2023). To investigate their involvement in wheat microspore embryogenesis, we identified more than 1500 differentially expressed TFs (DETFs) at each developmental transition in Nanda. Of these, 68% were upregulated during the initial transition from Day 0 to Day 7 (NS_D7), and 61% during the second transition from Day 7 to Day 21 (ND21_D7) (Additional file S4; Fig. 5A).

The DETFs belonged to over 30 TF families. In the ND7_D0 stage, major upregulated families included the Basic helix-loop-helix TF family (bHLH), WRKY TF family, Ethylene-responsive TF (ERF), Homeodomain-leucine zipper TF (HD-ZIP), Basic leucine zipper TF family (bZIP), GRAS TF family, and Auxin response factors (ARF) (Fig. 5B). By ND21_D7, an even broader range of TF families showed upregulation, including notable expansion in the TIFY family, where 13 of 30 members were upregulated, none of which were induced at the earlier stage. TF families, including bHLH, bZIP, ERF, GRAS, and WRKY have been reported to show embryo-specific enrichment during wheat zygotic-embryo development (Xiang et al. 2019). Transcription factors from multiple families, including bHLH, ERF, and WRKY, have also been reported to be expressed during barley microspore embryogenesis (Nowicka et al. 2024).

We further identified master regulatory TFs known to drive embryogenic processes (Yuan et al. 2023) (Fig. 5B). Members of the WRINKLED /PLETHORA /AINTEGUMENTA-like (WRI/PLT/AIL) TFs from APETALA2 (AP2) TF family, WUSCHEL-related homeobox (WOX) genes from the homeobox TF family, and Growth-regulating factors (GRFs) showed consistent upregulation across both developmental transitions in Nanda (ND7_D0 and ND21_D7). In contrast, TFs such as ABA INSENSITIVE3 (ABI3), NF-YB and ﻿RWP-RK DOMAIN − CONTAINING PROTEIN (RKD) were primarily upregulated at ND7_D0 but downregulated at ND21_D7. The VIVIPAROUS1/ABI3-LIKE (VAL) TFs displayed opposite regulation: downregulated early (ND7_D0) and upregulated later (ND21_D7), supporting their transcriptional repressor roles during embryogenesis (Baile et al. 2022; Yuan et al. 2023).

To understand the basis of Nanda’s higher embryogenic efficiency compared to Sadash, we analyzed DETFs from genotype comparisons across the three developmental stages (NS_D0, NS_D7, and NS_D21). We identified 440, 589 and 454 DETFs in Nanda relative to Sadash, with 66%, 67% and 61% of them upregulated, respectively (Additional file S4; Fig. 5C and D). Commonly enriched TF families among the upregulated DETFs at NS_D0 and NS_D7 included bZIP, WRKY, and the bHLH families. At NS_D7, additional upregulated TF families included HD-ZIP, ARF and ERF, while GRAS was prominent at NS_D21. Regarding master regulators, TFs such as WOX, ABI3, WRI/PLT/AIL, and NY-FB, were significantly upregulated at early stages (NS_D0 and NS_D7) but downregulated at NS_D21. GRF was upregulated early but not differentially expressed on Day 21; whereas VAL showed an inverse trend, downregulation on Day 0 but upregulation by Day 21. While fewer DETFs were identified between genotypes compared to within-stage transitions in Nanda, those detected may represent key regulators responsible for Nanda's superior embryogenic potential. In summary, the identification of stage-specific master regulators, including WRI/PLT/AIL, WOX, ABI3, and GRF TFs, highlights transcriptional networks likely driving successful embryogenesis in Nanda and points to potential targets for improving recalcitrant genotypes like Sadash.

### Genomic regions enriched in DEGs

Genomic structural variations or rearrangements are strongly associated with differences in gene expression and, consequently, influence diverse biological processes. Identifying genomic regions where genes are co-expressed or co-localized can provide insights into how such variations contribute to these biological processes. To explore this, we used PREDA to scan the genome for genomic regions enriched with DEGs in the comparison of Nanda and Sadash, highlighting the basis of Nanda’s higher embryogenic efficiency compared to Sadash.

**Fig. 6:**
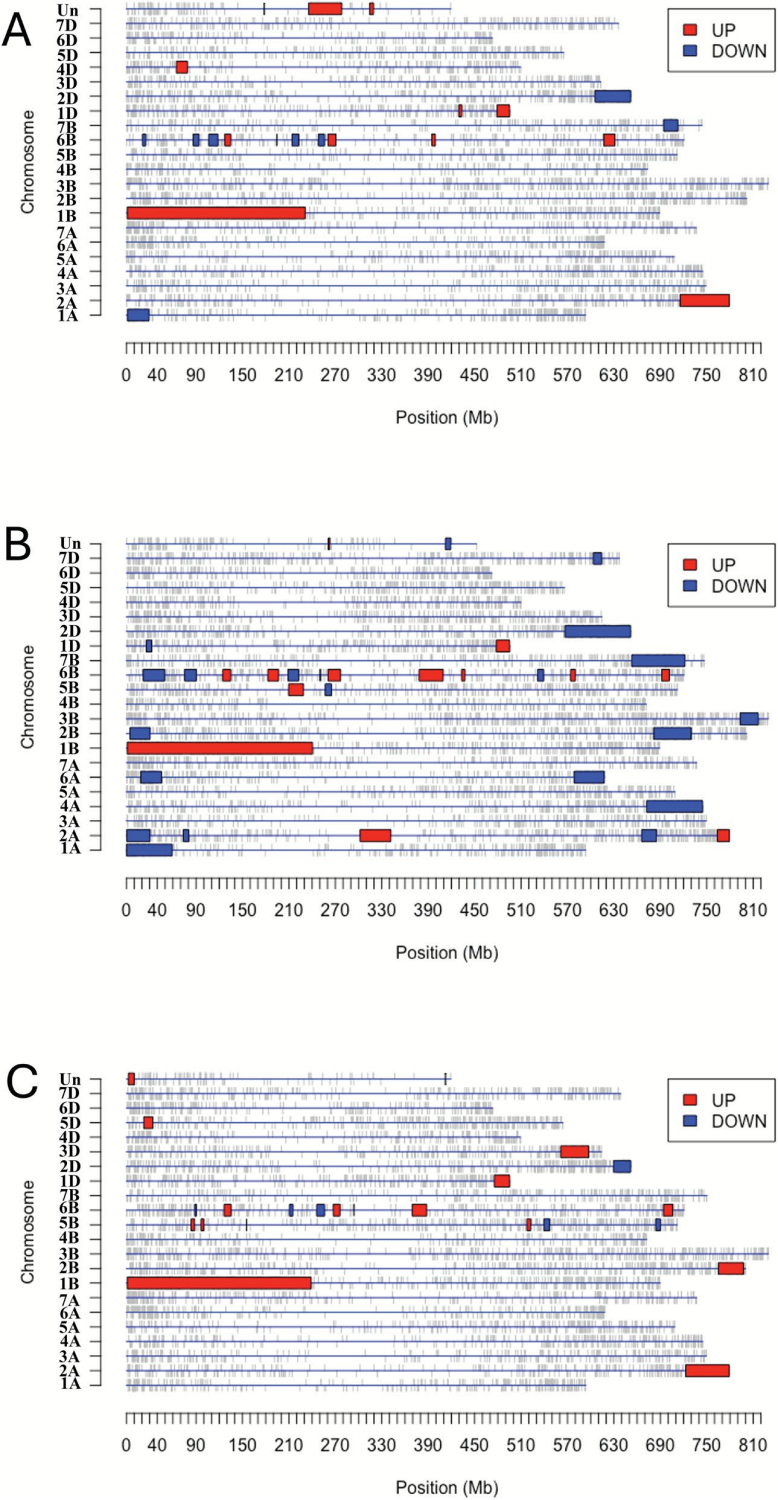
Genome plots illustrating genomic regions enriched with DEGs. **A** Genome plot from the DEGs between Nanda and Sadash on Day 0 (NS_D0); **B** genome plot from the DEGs between Nanda and Sadash on Day 7 (NS_D7); (C) Genome plot from the DEGs between Nanda and Sadash on Day 21 (NS_D21); Un on *Y*-axis refers to unanchored scaffolds in the wheat reference genome (IWGSC v1.1). Blue boxes represent regions enriched with down-regulated genes and red boxes represent regions enriched with up-regulated genes. FDR-corrected *p*-value < 1e–05 is used to define the significantly enriched genomic regions

Comparative analysis of DEGs across the three developmental stages (NS_D0, NS_D7, and NS_D21) revealed striking patterns of genomic enrichment. In each stage, more than 20 genomic regions enriched with DEGs were identified, with the most prominent region located on Chr 1B, spanning a ~ 230 Mbp region that is consistently enriched with upregulated DEGs and shared across all three stages in Nanda vs. Sadash comparisons (Fig. 6A–C**, **Additional file S5). Additional enriched genomic regions with upregulated DEGs were distributed across multiple chromosomes. Although most of these regions were stage specific, 24 out of 40 regions were located on chromosomes from the B sub-genome, with a strong representation on Chr 6B. Similarly, more than 30 genomic regions enriched with downregulated DEGs were detected across all three stages, with 23 of these located in the B sub-genome, again with a concentration on Chr 6B (Fig. 6A–C). Notably, the commonly enriched genomic regions on Chromosomes 1B, 1D, 2A, 2D, and 6B across all three stages likely represent genomic hotspots where structural variations or rearrangements distinguish Nanda from Sadash, contributing to their contrasting embryogenic responses, with additional enriched genomic regions linked to stage-specific processes.

### Discovery of androgenesis candidate genes using machine learning

While DEG- and GO-based analyses revealed regulatory processes, TFs, and genomic regions associated with androgenesis, we further sought to identify specific candidate genes that may serve as key regulators of embryogenic efficiency. To achieve this, we applied machine learning algorithms to identify key genes serving as discriminative features in microspore embryogenesis, using the R package MLSeq (Goksuluk et al. 2019). In this framework, all datasets from the low-embryogenic genotype Sadash were used as the reference class, allowing robust discrimination of the highly embryogenic Nanda.

Model training and evaluation were conducted using two parameter sets: fivefold cross-validation repeated twice, and fivefold cross-validation repeated ten times. Among the models tested, the Voom-based Nearest Shrunken Centroids model (vmodel) demonstrated the highest accuracy, sensitivity, and specificity and was therefore employed for feature selection (Additional file 1: Fig. S6A and S6B). From a total of 62,692 expressed genes, only two genes, TRAESCS1D02G454400 and TRAESCS6A02G408200, were predicted as the most informative discriminators between Nanda and Sadash. Of these, TRAESCS1D02G454400 was significantly upregulated in Nanda across all three stages (NS_D0, NS_D7, and NS_D21, Additional file 1: Fig. S6C and S6D). By contrast, TRAESCS6A02G408200 was consistently downregulated in Nanda.

TRAESCS1D02G454400 is an ortholog of Arabidopsis genes AT3G44750 and AT5G03740, both belonging to the histone deacetylase family (HD2A/HDT1 and HD2C/HDT3). Histone deacetylases (HDACs) are essential epigenetic regulators that remove acetyl groups from histone tails, therefore, regulating chromatin organization that influences the expression of genes associated with diverse developmental processes, including DNA repair and replication, metabolism, cytoskeletal dynamics, cell signaling and stress adaptation (Liu et al. 2014; Cui et al. 2023). Based on sequence homology and functional characteristics, plant HDACs are classified into three major families: the RPD3/HDA1 family, the plant-specific HD2 family, also known as the HD-tuin family, and the sirtuin family (Hollender and Liu 2008). The RPD3/HDA1 family is present in all eukaryotes, further divided into three classes, and it consists of 12 members in *Arabidopsis thaliana*, including HDA6, HDA17, and HDA19 (Pandey et al. 2002). The plant-specific HD2 family or HD-tuin family comprises four members in *Arabidopsis thaliana*, namely HD2A/HDT1, HD2B/HDT2, HD2C/HDT3 and HD2D/HDT4; while only two members, SRT1 and SRT2, are in the sirtuin family (Pandey et al. 2002; Hollender and Liu 2008).

Homologue to HD2A was upregulated, while homologues to HDA6 and HDA19 were downregulated in an early transcriptomic study of wheat microspore embryogenesis induction (Seifert et al. 2016). HDA6 and HDA19 showed a redundant role in the repression of the embryonic program after germination in *Arabidopsis thaliana* (Tanaka et al. 2007), and both were considered as part of the HDAC complex, along with HDA17, in the HDAC inhibitor trichostatin A (TSA) promoted microspore embryogenesis (Li et al. 2014). On the other hand, HD-tuin family members HDT1, HDT2 and HDT3 are involved in rDNA repression to regulate embryo development, with silencing of HDT2 expression resulting in seed abortion (Wu et al. 2000; Zhou et al. 2004; Luo et al. 2022). The differential expression of HDAC genes during embryo development, including microspore embryogenesis, reflects the functional diversity of HDAC family members and the dynamic epigenetic regulation that accompanies embryogenesis. Collectively, the convergence of differential expression, genomic enrichment, and machine learning predictions strongly supports TRAESCS1D02G454400 as a potential androgenesis marker gene in wheat, providing a promising target for functional validation and potential use in breeding strategies to enhance DH production efficiency.

The second candidate, TRAESCS6A02G408200, is a high-confidence gene annotated in the IWGSCv1.1 reference with a single transcript, but it has no known orthologs or paralogs and encodes an uncharacterized protein. Limited expression data indicate activity in wheat endosperm, aleurone layer, transfer cells, leaves, shoots, roots, spikes, and grains, but no additional information regarding protein structure, domains, or functional roles is currently available.

## Discussion

### Systematic transcriptomic reprogramming defines Nanda microspore embryogenesis

Microspore embryogenesis in wheat represents a remarkable developmental reprogramming process in which haploid microspores are redirected from the pollen developmental pathway toward embryogenesis in response to stress treatments, such as cold shock (Ferrie and Keller 1995; Kasha et al. 2001; Wang et al. 2019). In hexaploid wheat, this transition unfolds in two distinct phases: (1) the embryogenesis initiation phase (D0 to D7), during which microspores acquired embryogenic competence from cold treatment undergo cellular reprogramming leading to the initiation of embryogenesis, and (2) the embryogenic development phase (D7 to D21), characterized by active cell proliferation, tissue organization, and embryo differentiation. Our comprehensive transcriptomic profiling of the highly embryogenic wheat cultivar Nanda revealed more than 20,000 differentially expressed genes (DEGs) across these transitions, enriched in over 200 biological processes, reflecting a highly orchestrated molecular program that shifts microspores from a gametophytic to an embryogenic fate. The scale and directionality of these changes highlight the interdependence of transcriptional, epigenetic, metabolic, and structural remodeling processes, which collectively form the foundation for successful reprogramming (Pauls et al. 2006).

### Stress signaling, structural dynamics, and metabolic reprogramming during microspore embryogenesis

The induction of microspore embryogenesis in wheat is a complex process that integrates stress perception, signaling networks, structural reorganization, and metabolic reprogramming. Stress treatments are a prerequisite for inducing microspore embryogenesis in cereals and Brassicaceae. Various treatments have been utilized, including heat or cold shock, carbohydrate starvation, chemical induction, and mechanical stresses (Ferrie and Keller 1995; Touraev et al. 1996; Zheng 2003; Shariatpanahi et al. 2006; Soriano et al. 2008; Wang et al. 2019). The application of these treatments alone or in combination acts as triggers that disrupt the normal pollen developmental program and initiate embryogenesis. However, these treatments also lead to the generation of reactive oxygen species (ROS), creating oxidative stress. The ability of microspores to mitigate oxidative damage while maintaining ROS as signaling molecules is critical for embryogenic induction (Testillano 2019). In Nanda, the glutathione metabolic process was prominently upregulated during the induction stage, with dozens of glutathione *S*-transferase (GST) genes activated. These enzymes not only detoxify ROS but also participate in redox signaling, positioning them as crucial regulators of embryogenic competence (Dixon et al. 2009). Activation of GST genes associated with androgenesis induction has been reported previously in other crops, such as barley (*Hordeum vulgare*), triticale (× *Triticosecale* Wittm.) and wheat (*Triticum aestivum*), through gene expression studies or microarray analysis (Maraschin et al. 2006; Jacquard et al. 2009; Muñoz-Amatriaín et al. 2009; Sánchez-Díaz et al. 2013; Żur et al. 2014). A recent transcriptomic study of barley microspore embryogenesis during the early stages of induction, including Day 0, Day 2 and Day 5, using a responsive genotype, also showed up-regulation of various GST genes (Bélanger et al. 2018). This suggests that targeted manipulation of ROS-scavenging pathways could be a promising strategy to enhance embryogenesis in recalcitrant wheat genotypes.

Concurrently, hormonal signaling networks play indispensable roles in coordinating stress perception with developmental reprogramming during microspore embryogenesis. Endogenous auxin synthesis and polar transport are required for stress-induced microspore embryogenesis in barley(Pérez-Pérez et al. 2019). Hormonal homeostasis, including proper endogenous/exogenous auxin balance, favors androgenesis in triticale (Żur et al. 2015b; Juzoń-Sikora et al. 2023). A series of biological processes related to hormone-mediated signalling pathways and cellular responses to hormone stimulus is enriched from upregulated genes throughout Nanda microspore embryogenesis. Pathways related to auxin, ethylene, and abscisic acid (ABA) were consistently upregulated across developmental stages. Their dynamic interplay suggests a crosstalk mechanism where auxin regulates cell division and polarity, ethylene mediates stress adaptation, and ABA contributes to stress resilience and embryo maturation (Rodríguez-Sanz et al. 2015; Żur et al. 2015a). Beyond these, a wide spectrum of signaling pathways, responding to abiotic stresses, nutrient availability, and inorganic or organic stimuli, was enriched, reflecting a complex feedback system that integrates external cues with internal developmental programs. The interplay of these pathways may provide multiple intervention points to enhance or synchronize embryogenic responses.

Reprogramming of microspores is marked by a switch from asymmetric nuclear division typical of pollen development to symmetric division, a hallmark of embryogenic entry (Pauls et al. 2006). This switch is supported by extensive reorganization of the cytoskeleton, including actin filaments and microtubules, which regulate cell polarity, division planes, and intracellular organization (Gervais et al. 2000; Takemoto and Hardham 2004; Seguí-Simarro and Nuez 2008). Microtubule configuration and cytoskeleton reorganization were visualized in canola microspore embryogenesis utilizing immunocytochemistry and confocal laser scanning microscopy (Dubas et al. 2011). Microtubule fragmentation and depolymerization, and subsequent microtubule reconfiguration, are essential for the induction of wheat microspore embryogenesis after mannitol and n-butanol treatments (Dubas et al. 2021). During the induction stage, cytoskeleton organization and microtubule-based processes were strongly upregulated in Nanda, providing mechanical support for cell division and polarization. In parallel, the cell wall undergoes dramatic remodeling. During the induction phase, rigid pollen-specific walls are replaced by more flexible, callose-rich but cellulose-deficient walls, allowing for rapid proliferation and multicellular structure formation (Solís et al. 2008; Parra-Vega et al. 2015; Barnes and Anderson 2018; Corral-Martínez et al. 2019; Rivas-Sendra et al. 2019). This dynamic remodeling is later reversed during the embryo development phase, reflecting the restoration of structural integrity and preparation for differentiation. This cyclical cell wall remodeling represents a structural checkpoint in the transition from gametophytic to embryogenic fate.

Metabolic reprogramming complements these structural and signaling changes. At the induction stage, glutamine metabolism is strongly activated, providing nitrogen, energy, and signaling molecules to support rapid cell division. Glutamine has been shown to be beneficial for somatic embryogenesis, and exogenous glutamine has been shown to enhance embryogenesis in camelina, highlighting its importance as a metabolic driver (Ogita et al. 2001; Ferrie and Bethune 2011; Carlsson et al. 2017; Daniel et al. 2018; Lee et al. 2023). As embryogenesis advances, phenylalanine metabolism predominates, feeding into the phenylpropanoid pathway that supplies lignin, flavonoids, and hormone precursors necessary for cell wall reinforcement, stress tolerance, and embryo differentiation (Barros and Dixon 2020). Starch accumulation is important for pollen maturation and viability, therefore, starch biosynthesis is usually considered a marker of the pollen development pathway in cereal crops, while starch breakdown indicates the induction of microspores (Soriano et al. 2013; Liu et al. 2021). The downregulation of starch biosynthesis during the induction phase and double fertilization-related pathways during the embryogenesis phase in Nanda confirms the developmental switch from the gametophytic to embryogenic fate. Collectively, the integration of stress perception, hormonal signaling, cytoskeletal and cell wall remodeling, and targeted metabolic shifts establishes the cellular environment necessary for successful microspore embryogenesis in Nanda, highlighting multiple avenues for improving embryogenic efficiency in wheat breeding programs.

### Epigenetic plasticity and genomic context shape genotype-specific outcomes

Epigenetic regulation emerged as one of the most dynamic processes in Nanda microspore embryogenesis. During the induction stage (ND7_D0), nucleosome assembly, chromatin organization, and chromatin remodeling were among the most upregulated processes, establishing a permissive chromatin state that allows broad transcriptional flexibility. Nucleosomes, the fundamental units of chromatin consisting of DNA wrapped around histone octamers (two copies each of H2A, H2B, H3, and H4), act as key regulators of transcriptional activity by modulating DNA accessibility (Kornberg and Lorch 2020). Dynamic chromatin organization provides the flexibility needed to rapidly and reversibly reprogram gene expression in response to stress (Hemenway and Gehring 2023; Jo and Nodine 2024; Li et al. 2024). Such chromatin-based regulation has been widely implicated in microspore embryogenesis across species, including *Brassica napus* and *Hordeum vulgare* (El-Tantawy et al. 2014; Nowicka et al. 2024; Pérez-Pérez et al. 2025). Notably, these processes are progressively downregulated during the later embryogenic phase (ND21_D7), reflecting a temporal transition from permissive and flexible transcriptional states during induction to more stable chromatin configurations that sustain differentiation programs. Comparisons with the low-embryogenic cultivar Sadash revealed that stage-specific regulation of chromatin remodeling, DNA methylation, and nucleosome dynamics was more finely tuned in Nanda.

Through machine learning, TRAESCS1D02G454400, a histone deacetylase (HDAC) ortholog, was identified as a strong biomarker of embryogenic competence. HDACs are central players in chromatin remodeling, transcriptional regulation, and developmental reprogramming (Liu et al. 2016; Cui et al. 2023). Genome-wide scans further highlighted DEG-enriched regions on chromosomes 1B, 1D, 2A, 2D, and 6B, with TRAESCS1D02G454400 residing within a highly enriched DEG cluster on Chr 1D. This points toward an interaction between structural genomic variation and epigenetic regulation as a mechanistic basis for the contrasting embryogenic responses in Nanda vs. Sadash. In wheat’s hexaploid genome, homoeolog expression provides additional resolution to this regulatory landscape. Differential expression biases across the A, B, and D subgenomes indicate that epigenetic mechanisms and chromatin accessibility not only regulate global transcription but also shape subgenome-specific contributions to embryogenic potential. In particular, the B subgenome harbored a disproportionately high number of enriched DEG regions, consistent with its major role in modulating genotype-specific embryogenic outcomes.

Transcription factors (TFs) act as central regulators coordinating the structural, metabolic, and epigenetic changes. In Nanda, key embryogenesis-related TFs, including WOX, PLT/AIL, ABI3, and NF-Y orthologs, were differentially expressed across developmental stages. WUS and its close homologs WOXs have been identified as important factors during somatic embryogenesis and WOX genes show induced expression during canola microspore embryogenesis (Soriano et al. 2013; Horstman et al. 2017). BBM/PLT/AILs promote early embryo development through the induction of meristematic potential, are key players in somatic embryogenesis, and up-regulated during wheat microspore embryogenesis induction (Seifert et al. 2016; Horstman et al. 2017; Kerstens et al. 2024). Homologues of BBM/AIL and WOX were also expressed during the induction of barley microspore embryogenesis, and were subsequently identified among a set of potential marker genes for effective reprogramming of barley microspores (Bélanger et al. 2018; Nowicka et al. 2024). NF-Y TF family genes are required in early embryogenesis and show induced expression in barley and triticale microspore embryogenesis (Fornari et al. 2013; Żur et al. 2014; Nowicka et al. 2024). ABI3 plays a critical role during somatic embryogenesis and has been proposed as a molecular marker for early microspore embryogenesis in canola (Malik et al. 2007; Smertenko and Bozhkov 2014). The expression patterns of these TFs are consistent with their known roles in meristem induction, early embryo patterning, and suppression of pollen-specific programs, indicating a direct regulatory link between chromatin accessibility and transcriptional cascades driving embryogenic reprogramming. Members of the B3 transcription factor family, VIVIPAROUS/ABI3-LIKE genes (VALs, or HSI2 and HSL1) have been shown as transcriptional repressors for gene silencing (Duarte-Aké et al. 2019; Yuan et al. 2023). Downregulation of VAL orthologs during the induction stage likely facilitates activation of embryogenesis-related genes. These TFs represent potential molecular markers for early embryogenic competence, providing targets for functional validation or molecular-assisted selection in wheat breeding.

## Conclusions

Genotypic recalcitrance remains a major barrier to the widespread application of DH technology in bread wheat and other cereals. By dissecting the expression dynamics of two contrasting wheat genotypes, this study uncovers the multilayered regulatory networks that govern microspore embryogenesis. We show that successful and highly efficient embryogenesis in Nanda is driven by coordinated regulation of stress perception, ROS homeostasis, hormonal signaling, cytoskeletal and cell wall remodeling, and metabolic reprogramming, with epigenetic and genomic regulation serving as the dominant driver. The identification of stage-specific DEG-enriched genomic regions, subgenome-specific expression patterns, and predictive biomarkers such as TRAESCS1D02G454400 highlights the intricate interplay between chromatin dynamics, transcriptional regulation, and structural genomic variation in shaping embryogenic competence. The differential regulation observed between Nanda and Sadash further underscores the role of finely tuned epigenetic and subgenomic contributions in determining genotype-specific outcomes.

From an applied perspective, these findings open opportunities to harness candidate regulators, including HDACs, master TFs, ROS-scavenging enzymes, and metabolic genes, for marker-assisted selection, CRISPR/Cas-mediated genome editing, or chemical modulation to improve embryogenic efficiency. Moreover, DEG-enriched genomic regions and homoeolog expression biases provide a blueprint for subgenome-targeted breeding strategies, accelerating DH production pipelines. Collectively, this integrated framework deepens our understanding of developmental plasticity in wheat microspore embryogenesis and paves the way for extending such strategies to other recalcitrant cereal crops. By bridging fundamental biology with applied breeding, these insights contribute directly to accelerating variety development and addressing the challenges of future agricultural production.

While this study provides a comprehensive transcriptome-based framework for understanding genotype-specific microspore embryogenesis in wheat, we note that the conclusions are based on correlative gene expression analyses. Functional validation of the identified candidate regulators, pathways, and biomarkers through genetic, molecular, or biochemical approaches will be essential to establish causal relationships and confirm their roles in embryogenic competence. Future studies integrating targeted gene perturbation, epigenetic profiling, and physiological assays will be critical to translating these transcriptomic insights into validated tools for wheat improvement.

## Supplementary Information

Below is the link to the electronic supplementary material.

Supplementary file1 FigS1 (PNG 373 KB)

Supplementary file2 FigS2 (PNG 8951 KB)

Supplementary file3 FigS3 (PNG 3889 KB)

Supplementary file4 FigS4 (PNG 98981 KB)

Supplementary file5 FigS5 (PNG 36297 KB)

Supplementary file6 FigS6 (PNG 4780 KB)

Supplementary file7 Additional File S1_wheatDH_DEG_MercatorAnnotation (XLSX 5969 KB)

Supplementary file8 Additional File S2_wheatDH_triad_exp_5TPM (XLSX 6523 KB)

Supplementary file9 Additional File S3_wheatDH_DEG_GOBP_enrichList (XLSX 193 KB)

Supplementary file10 Additional File S4_wheatDH_DETF_MercatorAnnotation (XLSX 369 KB)

Supplementary file11 Additional File S5_wheatDH_PREDA_NS_DEG (XLSX 67 KB)

## Data Availability

The raw sequencing data have been submitted to the NCBI Sequence Read Archive with the GEO accession number GSE310687.
