# Supplementary figures and images for: Transcriptomic analyses reveal regulatory plasticity and metabolic reprogramming underlying genotype-specific microspore embryogenesis in wheat
Source: Plant Cell Rep. 2026 Feb 4;45(2):51. doi: 10.1007/s00299-026-03731-x (PMC12872716; doi:10.1007/s00299-026-03731-x)

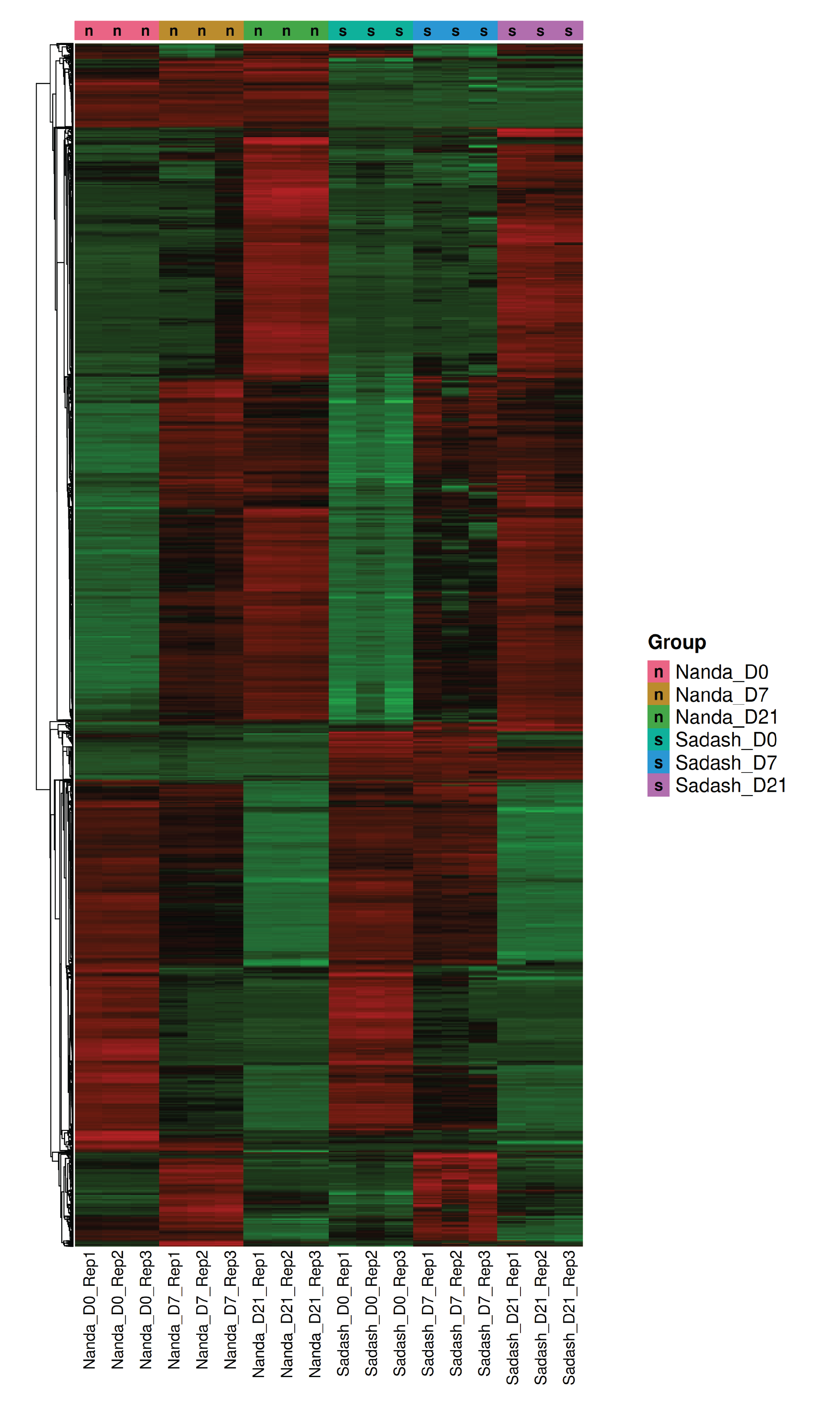

Supplement: Supplementary file 1 — Supplementary file1 FigS1 (PNG 373 KB) [file 299_2026_3731_MOESM1_ESM.png]

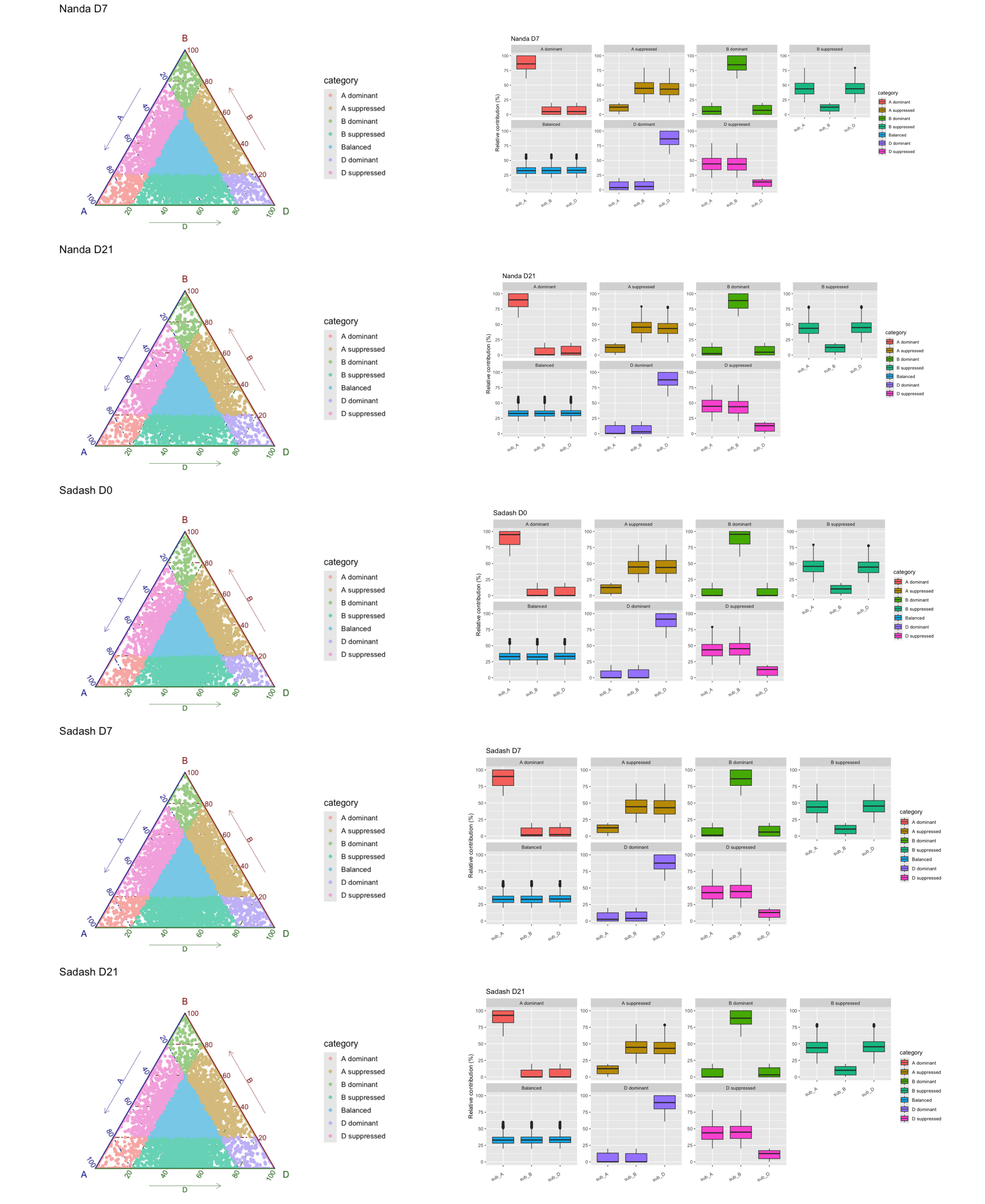

Supplement: Supplementary file 3 — Supplementary file3 FigS3 (PNG 3889 KB) [file 299_2026_3731_MOESM3_ESM.png]
